# Knowledge, Attitudes, and Practices Regarding HIV/AIDS Among Adolescents in Internally Displaced Persons (IDP) Camps in Mogadishu, Somalia: A Descriptive Cross-Sectional Study

**DOI:** 10.1155/arat/5911447

**Published:** 2025-10-06

**Authors:** Najib Isse Dirie, Mulki Mukhtar Hassan, Amal Naleye Ali, Mohamed Mustaf Ahmed, Jaweriya Bashir Ahmed, Abdullahi Hassan Elmi, Hamza Mohamed Abdullahi, Liban Hassan Mohamed

**Affiliations:** ^1^Department of Urology, Dr. Sumait Hospital, Faculty of Medicine and Health Sciences, SIMAD University, Mogadishu, Somalia; ^2^Dr. Sumait Hospital, Faculty of Medicine and Health Sciences, SIMAD University, Mogadishu, Somalia; ^3^Faculty of Medicine and Health Sciences, SIMAD University, Mogadishu, Somalia; ^4^Department of Nursing, Dr. Sumait Hospital, Faculty of Medicine and Health Sciences, SIMAD University, Mogadishu, Somalia; ^5^Department of Research, Federal Ministry of Health Somalia, Mogadishu, Somalia; ^6^Institute of Climate and Environment (ICE), SIMAD University, Mogadishu, Somalia

**Keywords:** adolescents, attitudes, HIV/AIDS, internally displaced persons, knowledge, practices, Somalia

## Abstract

**Background:**

HIV/AIDS remains a major global health challenge worldwide. Adolescents, particularly those in displaced populations, are highly vulnerable to HIV infection. This study aimed to assess the knowledge, attitudes, and practices (KAP) regarding HIV/AIDS among adolescents in internally displaced persons (IDP) camps in Mogadishu, Somalia.

**Methods:**

We conducted a descriptive cross-sectional study of adolescents aged 13–19 years living in IDP settlements in Mogadishu's Kahda and Deynile districts. Data were collected using a validated questionnaire. We constructed composite KAP indices using prespecified cutoff points, summarized outcomes descriptively, and compared groups by age and gender using *χ*^2^ tests and one-way ANOVA, with ethical approval and assent/consent procedures in place.

**Results:**

This study included 440 adolescents with a mean age of 15 ± 2 years, 68% of whom were female. The majority (69%) demonstrated poor knowledge of HIV/AIDS, with common misconceptions about transmission, such as 75% believing that mosquito bites and 85% believing that sharing meals could transmit HIV infection. Negative attitudes toward people living with HIV/AIDS were prevalent, with 84% displaying stigmatizing views. For instance, 81% believed that HIV-positive students should not attend school, and 89% were unwilling to buy vegetables from an HIV-positive vendor. HIV testing rates were extremely low at 0.45%, and only 2.1% of sexually active participants reported condom use. Participants were primarily from the Deynile and Kahda districts, with significant representation from the Southwest State and Hirshabelle. No significant differences in knowledge and attitude scores were observed between the sex or age groups.

**Conclusion:**

Adolescents in IDP camps in Mogadishu demonstrated poor knowledge, stigmatizing attitudes, and risky HIV/AIDS practices. These findings highlight the urgent need for culturally sensitive educational programs, improved access to testing and counselling services, and community-based stigma reduction interventions for this vulnerable population.

## 1. Introduction

Since the early 1980s, human immunodeficiency virus (HIV) and acquired immunodeficiency syndrome (AIDS) have emerged as significant global health challenges, impacted millions of lives, and posed substantial barriers to public health and socioeconomic development worldwide [1, 2]. As of 2024, the United Nations Program on HIV and AIDS (UNAIDS) reported that approximately 39 million people are living with HIV, with 1.3 million new infections and 630,000 AIDS-related deaths occurring globally, most of whom are adults aged ≥ 15 years [3]. Adolescents represent a particularly vulnerable group, with approximately 1 million individuals aged 15–19 years living with HIV globally in 2023, and a significant majority (84%) residing in sub-Saharan Africa. Beyond sub-Saharan Africa, the highest number of HIV-positive adolescents has been found in Asia and Latin America. Among adolescents living with HIV, 85% were girls in Eastern and Southern Africa and 81% in West and Central Africa. In contrast, in East Asia and the Pacific, 71% of adolescents with HIV are boys [4].

Adolescent-focused HIV indicators remain underreported in Somalia, particularly among displaced youth, necessitating targeted evidence to guide adolescent-friendly services [5, 6]. Adolescents, particularly aged 15–24, are highly vulnerable to HIV infection because of early sexual activity, exploitation, and insufficient access to accurate information [7–10]. The limited availability of youth-friendly services further compounds this risk, contributing to higher rates of risky behaviors and sexually transmitted diseases, including HIV/AIDS and unintended pregnancies [11]. Persistent gender disparities, discrimination, and poverty hinder adolescent girls from achieving economic autonomy and making informed sexual decisions, thereby exposing them to ongoing emotional and physical risks. This significantly increases their vulnerability to HIV infection, especially in sub-Saharan Africa, where the HIV prevalence among adolescent girls is over three times higher than that among young men, highlighting the urgent need for targeted interventions for HIV prevention and treatment [12–14].

The global displacement crisis has affected over 100 million people by 2023, who are facing severe health challenges. Somalia has the world's lowest health indicators and over three decades of civil war and instability, resulting in the internal displacement of over 3.7 million people, with many resettling in more than 2400 substandard internally displaced persons (IDP) sites [15]. Ongoing conflicts, along with droughts, floods, and locust infestations, have perpetuated food crises, poverty, and inadequate infrastructure [15]. Various factors contribute to the association between displacement and HIV infection. For example, unstable housing post-displacement can hinder access to HIV care, increasing the vulnerability of displaced individuals to the virus [16, 17]. They also face heightened vulnerability due to disrupted social structures, economic hardship, sexual violence, substance abuse, inadequate healthcare services, and limited access to education [18]. During conflict and displacement, men may engage in opportunistic sexual encounters or seek sex workers. Conversely, women frequently experience sexual violence, coercion in transactional sex, and relationships for various reasons [12, 19, 20]. Therefore, displaced populations consistently need access to reproductive and sexual health services [21].

Globally, HIV/AIDS continues to pose a major public health challenge, affecting millions across all age groups. Sub-Saharan Africa remains the most impacted region, accounting for many HIV cases worldwide. Adolescents, especially girls, face heightened vulnerability due to early sexual debut, gender inequality, and limited access to reproductive health education and services [7]. Moreover, improving HIV knowledge can help reduce stigma against people living with HIV/AIDS, as stigmatizing attitudes are often rooted in misconceptions about HIV transmission and negative perceptions of those living with the virus [22]. By addressing the unique challenges faced by displaced individuals, particularly the youth, and implementing targeted strategies, Somalia can make significant strides in combating the HIV/AIDS epidemic in the country.

In Somalia, although the officially reported HIV prevalence is relatively low, the actual burden may be underestimated due to underreporting, stigma, and limited access to testing services. Adolescents living in IDP camps face even greater risks due to extreme poverty, disrupted education, lack of healthcare infrastructure, and displacement-related trauma. These overlapping vulnerabilities make HIV prevention, education, and care particularly challenging for this population. Despite the known risks, there is a critical gap in the research specifically examining the knowledge, attitudes, and practices (KAP) of HIV/AIDS among adolescents in Somali IDP camps in Somalia. Existing studies rarely capture the lived experiences of displaced youth in these settings. This study aims to fill this gap by providing context-specific data to inform targeted interventions that promote HIV awareness, prevention, and stigma reduction among this underrepresented and vulnerable population.

## 2. Methodology

### 2.1. Study Design and Setting

A descriptive cross-sectional study was conducted to assess the KAP regarding HIV/AIDS among adolescents residing in IDP camps in Mogadishu, Somalia. The study was conducted over 6 weeks (May 1–June 15, 2024) in IDP camps located in the Kahda and Deynile districts. Kahda hosts 864 verified IDP sites with 106,831 households and 594,612 individuals, facing challenges such as inadequate infrastructure and limited access to basic services [23]. Deynile accommodates 1115 verified IDP sites with 123,642 households and 653,057 individuals, with significant hardships, including high eviction risks and poor access to essential services [23].

### 2.2. Study Population and Sampling

The target population consisted of adolescents aged 13–19 years who had lived in the selected IDP sites for at least one year and were not pregnant. According to a 2023 OCHA report, IDP camps in Mogadishu are home to 1,247,669 individuals, with approximately 60% of the population being adolescent [23]. For this study, the adolescent population was estimated at 748,601 individuals (≈60% of 1,247,669). The sample size was calculated using the Yamane formula [24]:

(1) $$\begin{array}{c} n=\frac{N}{1+N\left(e^{2}\right)}, \end{array}$$

where *N* is the adolescent population and *e* = 0.05. The initial sample size was 400; to accommodate potential nonresponse, we increased the final target to 440. Participants were selected using convenience sampling with the assistance of the camp coordinators. Given the absence of a comprehensive sampling frame and security/logistical constraints in IDP settings, convenience sampling was used; this expedites recruitment in hard-to-reach, mobile populations but may introduce selection bias and limit generalizability of the findings.

### 2.3. Data Collection and Measures

Data were collected using a validated four-section structured questionnaire adapted from a previous study conducted in Cameroon [25].  Section 1 captures sociodemographic characteristics (age, gender, marital status, education, employment, district, federal state, reason for displacement, household size, family background, occupation, duration in current location, and monthly family income) and includes the confidentiality and voluntariness statement presented to participants; no personal identifiers were collected.  Section 2 assesses HIV knowledge (transmission, prevention, and misconceptions; 10 items).  Section 3 measures attitudes toward people living with HIV (five Likert-type items).  Section 4 captures practices (HIV testing, sexual activity, condom use, and drug use; five items). Composite KAP indices were constructed using prespecified cutoff points. Four trained data collectors conducted face-to-face interviews using the Google Forms application. The interviewers clarified any unfamiliar terms, and the interviews were conducted in private spaces within the camps to safeguard confidentiality.

The questionnaire was piloted with 50 adolescents from comparable IDP settings, and pilot participants were excluded from the main analysis to avoid contamination. Cronbach's alpha values indicated acceptable internal consistency: knowledge, 0.72; attitudes, 0.76; and practices, 0.63. Minor wording adjustments from the pilot study were incorporated before field deployment.

### 2.4. Data Analysis and Interpretation

Data were cleaned, coded, and analyzed using R (Version 4.4.0). Descriptive statistics were used to summarize the data: categorical variables as frequencies and percentages and continuous variables as means and standard deviations. Knowledge was scored from 0 to 10 (1 point per correct item) and categorized using Bloom's cutoffs into poor (< 60%, 0–5) and good (≥ 60%, 6–10) knowledge. Attitudes (five items) were scored 0–5 and categorized as negative (< 60%) or positive (≥ 60%). Although item responses were binary or ordinal, composite knowledge and attitude scores were treated as continuous variables to enable mean comparisons across groups. To explore differences by gender and age group (< 15, 15–17, > 17 years), one-way ANOVA was used for the composite scores. Statistical significance was set at *α* = 0.05 (two-sided). The final analytical dataset contained no missing data.

## 3. Results

### 3.1. Demographic and Socioeconomic Characteristics

**Figure 1:**
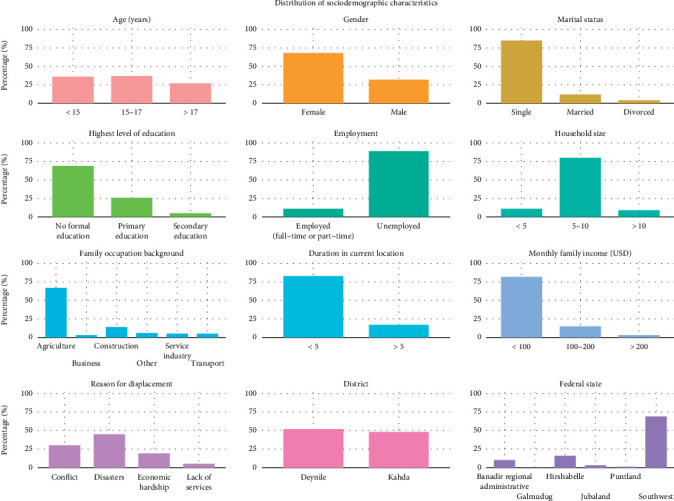
Distribution of sociodemographic characteristics of adolescents living in IDP camps in Mogadishu, Somalia (*n* = 440). Panels; age in years (< 15, 15–17, > 17); gender (female, male); marital status (single, married, divorced); highest level of schooling completed (no formal education, primary, secondary); current employment status at the time of interview (any paid work full- or part-time versus unemployed); household size as number of people living in the same dwelling (< 5, 5–10, > 10); family occupation background defined as the main livelihood of the parent or guardian (agriculture, business, construction, service industry/transport, other); duration in current location defined as the number of years the participant has lived in the present IDP site, not total time displaced or time in the district (< 5 years, > 5 years; sample mean 3 years, SD 2); monthly family income reported in US dollars (< 100, 100–200, > 200); primary reason for displacement (conflict or violence, natural disasters such as drought or flood, economic hardship, lack of basic services); district of residence within Mogadishu at survey (Deynile, Kahda); and federal state of origin (Banadir, Galmudug, Hirshabelle, Jubaland, Puntland, Southwest). Bars show percentages on a 0–100 percent *y*-axis. Percentages may not total 100 because of rounding.

This study included 440 adolescents from IDP camps in Mogadishu, Somalia. The mean age of the participants was 15 ± 2 years, with 36% under 15 years, 37% between 15 and 17 years, and 27% over 17 years (Figure 1). The sample was predominantly female, comprising 68% of the participants. The marital status distribution showed that 85% were single, 12% were married, and 4% were divorced. Education levels were notably low among participants, with 69% reporting no formal education, 26% having completed primary education, and only 5% having completed secondary education. Overall, 89% of the respondents were unemployed.

**Figure 2:**
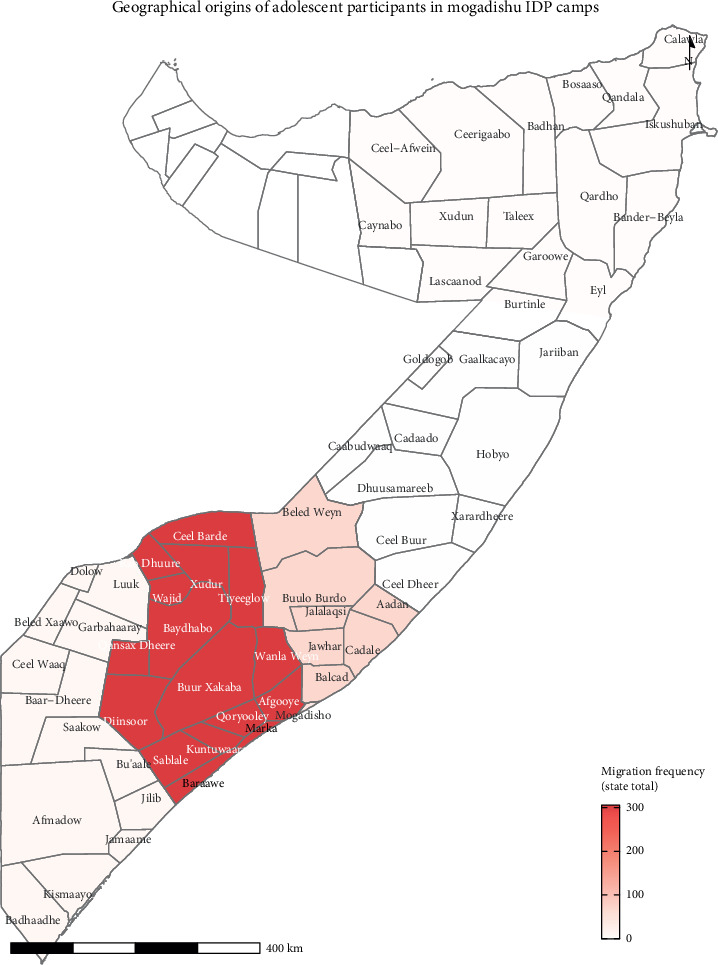
Geographical origins of adolescent participants in Mogadishu IDP camps.

The participants were primarily from the Deynile (52%) and Kahda (48%) districts, reflecting the major IDP settlements in Mogadishu. Regarding the Federal State distribution, most participants originated from the Southwest State (69%), followed by Hirshabelle (16%), Banadir Regional Administration (10%), Jubaland (3%), Puntland (1%), and Galmudug (less than 1%). The geographical distribution of the participants, as illustrated in Figure 2, indicates that a significant number of adolescents in the study migrated from districts within the Southwest State and Hirshabelle. The main reasons for displacement were natural disasters (45%), conflict or violence (30%), and economic hardship (19%); participants could select multiple reasons, so totals exceeded 100%. Most households comprised 5–10 members, with a mean household size of 7 ± 2 people. The predominant family occupation was agriculture, which accounted for 67% of the families. Economically, 82% of the families reported a monthly income below $100 USD, with a mean monthly income of $50 ± $66. The detailed distribution of the demographic and socioeconomic variables is presented in Table 1.

### 3.2. Knowledge About HIV/AIDS

**Figure 3:**
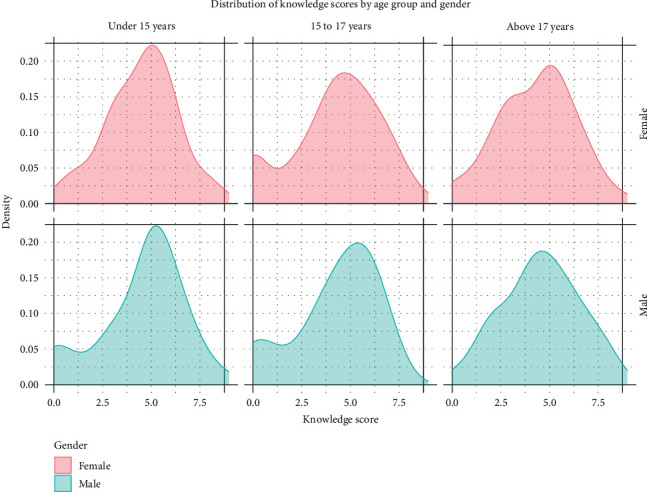
Distribution of knowledge scores by age group and gender.

Overall, knowledge about HIV/AIDS was poor, with 69% of the participants demonstrating poor knowledge and only 31% showing good knowledge, with a mean knowledge score of 4.40 (SD = 2.06). While 51% of the participants had heard of HIV/AIDS, many had significant misconceptions. For instance, 75% incorrectly believed that HIV could be transmitted through mosquito bites, and 85% thought HIV could be transmitted by sharing a meal with an infected person. Knowledge gaps were evident in prevention methods, with 70% unaware that having one faithful, uninfected partner could reduce HIV transmission risk, and 72% unaware that consistent condom use could reduce HIV risk (Table 2). However, the participants demonstrated better knowledge in some areas. Notably, 80% knew that HIV could be transmitted by sharing sharp objects, 76% were aware that HIV could be transmitted through breastfeeding, and 58% knew that HIV could be transmitted from mother to unborn child.  Figure 3 presents the score distributions by age and gender, and the inferential comparisons are summarized in Table 3.

### 3.3. Attitudes Toward HIV/AIDS

**Figure 4:**
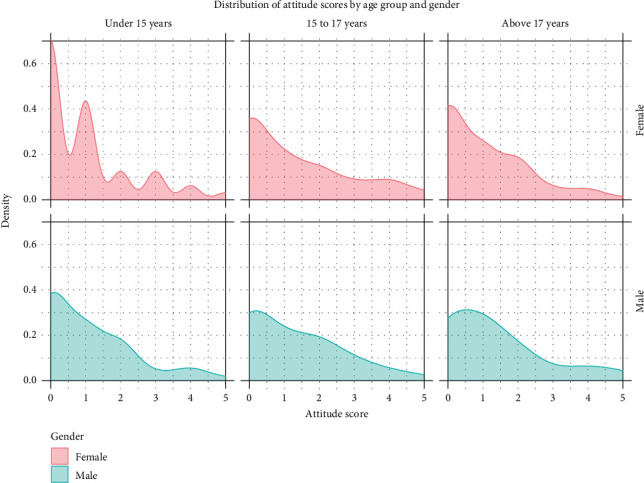
Distribution of attitude scores by age group and gender.

Most participants (84%) demonstrated negative attitudes toward people living with HIV/AIDS, with a mean attitude score of 1.17 (SD = 1.37). Stigmatizing attitudes were prevalent, with 66% of the respondents unwilling to care for a relative with HIV in their household (Table 4). Furthermore, 81% believed that HIV-positive students should not be allowed to continue attending school, and 80% believed that HIV-positive teachers should not be allowed to continue teaching. Stigma was also evident in social interactions, with 89% stating they would not buy fresh vegetables from a shopkeeper known to have HIV. Additionally, 71% reported that they wanted to keep it a secret if a family member became infected with HIV.  Figure 4 presents the attitude score distributions by age and gender, and the inferential comparisons are summarized in Table 3.

### 3.4. Practices Related to HIV/AIDS

HIV testing rates were extremely low, with only 0.45% (two out of 440 participants) reporting being tested for HIV. All participants were aware of their current HIV status. Regarding drug use, 5% reported using injectable drugs, and 39% of these individuals' shared needles or syringes. Among the 92 participants (21%) who reported being sexually active, only two (2.1%) reported using condoms during sexual intercourse (Table 5).

## 4. Differences in Knowledge and Attitude Scores by Gender and Age Groups

The ANOVA results (Table 3) indicated that there were no statistically significant differences in knowledge scores between men and women (*F* (1, df) = 0.222, *p* = 0.638) or across different age groups (*F* (2, df) = 0.548, *p* = 0.578). Similarly, no significant differences were found in attitude scores between the sexes (*F* (1, df) = 0.164, *p* = 0.685) or among age groups (*F* (2, df) = 2.067, *p* = 0.128). These findings suggest that gender and age did not significantly influence the knowledge and attitudes toward HIV/AIDS among the study participants.

## 5. Discussion

This study revealed a substantial deficit in knowledge about HIV/AIDS among the study participants, as indicated by the finding that 69% of the respondents had poor knowledge. Given the age profile of our sample (36% < 15 years; 37% 15–17 years), the very low formal employment observed (89% unemployed) is expected, as these ages typically coincide with schooling rather than labor market participation. In addition, the high proportion of those reporting no formal education (69%) indicates limited school attendance despite the predominance of school-age adolescents. This disconnect between age and education is consistent with the disruptions associated with displacement in Mogadishu IDP settlements.

Although 51% of participants reported being aware of HIV/AIDS, many harbored misconceptions about modes of transmission, such as the belief that mosquito bites (75%) and sharing meals with infected individuals (85%) could transmit HIV. These findings align with those of a previous study conducted among Nigerian adolescents [26], which also reported widespread misconceptions regarding HIV transmission among most participants. These misconceptions are not limited to African countries, as evidenced by a study conducted in Korea [27], which found that nearly 70% of adolescents believed that HIV could be transmitted through mosquito bites. Despite the participants' overall deficiency in HIV/AIDS knowledge, the majority correctly recognized sharing sharp objects and mother-to-child transmission as modes of HIV spread. These findings underscore the critical need to address persistent knowledge gaps among adolescents, particularly those related to HIV transmission.

Moreover, the study found that participants had limited awareness of preventative measures, such as the use of condoms and having a single faithful uninfected partner. Among the 92 participants (21%) who reported being sexually active, only two (2.1%) indicated using a condom during sexual intercourse. This suggests that even among those engaging in high-risk behavior, the knowledge and practice of prevention remain alarmingly low. This contrasts with a study conducted in Cameroon [25], which reported a relatively high rate of condom use among sexually active adolescents. The study also found a high prevalence of stigmatizing attitudes toward people living with HIV/AIDS among the participants. With 81% of participants believed that HIV-positive students should not be allowed to attend school, and 80% believed that HIV-positive teachers should not be allowed to teach. Furthermore, the study found that an overwhelming 89% of participants reported that they would refrain from purchasing produce from a vendor living with HIV/AIDS. Similar findings were echoed in a study conducted among high schools in South Africa [28], where approximately 50% of students expressed discriminatory attitudes toward people living with HIV/AIDS.

Participants exhibited significant stigmatizing attitudes toward caring for relatives with HIV. Sixty-six percent reported that they were unwilling to provide direct care and support for a family member living with HIV/AIDS. Moreover, 71% indicated that they would prefer to keep a family member's HIV status secret rather than openly acknowledge and support them if they were HIV-positive. These deeply rooted stigmatizing views toward people with HIV/AIDS reflect the substantial social discrimination that individuals living with the virus often face within this community, as indicated by national data from Somalia [29]. A study emphasized that HIV-related stigma is widespread in Somalia, leading to social isolation and discrimination, particularly in areas with limited access to education and employment opportunities [30]. Such attitudes can pose major barriers to HIV testing, treatment, and care, as people may fear the negative social consequences of disclosing their HIV status.

### 5.1. Limitations of the Study

While these findings provide valuable insights, it is important to acknowledge the limitations of this study for accurate interpretation. Reliance on self-reported data might have introduced response bias, particularly because the stigma associated with HIV/AIDS is prevalent. However, the anonymity of the questionnaire may have encouraged more honest responses. The sample size, limited to only two IDP camps in Mogadishu, may restrict the extent to which the findings can be applied to other displacement settings. Additionally, the study did not examine external factors such as access to health services, community support, and other sociocultural constraints, which are crucial in shaping attitudes toward and knowledge of HIV. Acknowledging these limitations is essential for accurately interpreting the findings and identifying the necessary areas for future research to further our understanding of these issues.

Despite these limitations, this study provides valuable insights for researchers and policymakers addressing HIV/AIDS challenges in similar contexts. These findings indicate the need for targeted interventions that address misinformation, promote testing, and reduce stigma. Low knowledge levels and high stigma directly inform the types of educational and behavioral strategies that must be prioritized.

To address these gaps, we recommend implementing targeted, culturally sensitive educational programs that correct misconceptions about HIV transmission and prevention. These should be delivered through school-based curricula, community workshops, and peer-led sessions. Involving community elders, religious leaders, and parents in these efforts can enhance their acceptance and outreach. To improve testing uptake, mobile HIV testing units and youth-friendly, confidential counselling services should be deployed in IDP camps, especially during health outreach campaigns. Health workers must be trained to ensure nonjudgmental communication and privacy. Mass communication campaigns using radio, social media, and storytelling formats can help normalize HIV-related discussions and reduce stigma. Additionally, integrating antistigma modules into school health education and promoting role models who advocate for HIV awareness can help shift harmful attitudes. These evidence-based and community-driven strategies are essential for reducing HIV vulnerability among displaced adolescents in Somalia.

## 6. Conclusion

Adolescents in Mogadishu's IDP camps show pronounced HIV knowledge gaps, pervasive stigma, extremely low testing uptake, and limited protective practices. These findings call for a focused package of adolescent-friendly, camp-based interventions: culturally tailored education to correct misconceptions, stigma-reduction activities embedded in schools and community forums, confidential youth-friendly HIV testing (including mobile outreach), reliable condom availability, and harm-reduction messaging for injecting risks. Partnering with community and religious leaders can extend reach and trust. Future work should prioritize the implementation and effectiveness studies of these interventions, complemented by qualitative research to identify barriers to testing and care. Humanitarian health information systems should routinely include adolescent-disaggregated indicators to track progress and guide adaptive programming in humanitarian settings.

## Figures and Tables

**Table 1:** Demographic and socioeconomic characteristics of the study population.

Variables	Frequency	Percentage
*Age (years)*		
< 15	158	36
15–17	162	37
> 17	120	27
Mean ± SD	15 ± 2	

*Gender*		
Female	298	68
Male	142	32

*Marital status*		
Single	372	85
Married	52	12
Divorced	16	4

*Highest level of education*		
No formal education	305	69
Primary education	114	26
Secondary education	21	5

*Employment*		
Unemployed	393	89
Employed (full-time or part-time)	47	11

*District*		
Deynile	227	52
Kahda	213	48

*Federal state of origin*		
Southwest	305	69
Hirshabelle	70	16
Banadir Regional Administrative	43	10
Jubaland	15	3
Puntland	5	1
Galmudug	2	0

*Reason for displacement*		
Natural disaster (e.g., drought, flood)	200	45
Conflict or violence in the area	133	30
Economic hardship or unemployment	85	19
Lack of access to basic services (e.g., healthcare, education)	22	5

*Household size (people)*		
< 5	47	11
10–5	352	80
> 10	41	9
Mean ± SD	7 ± 2	

*Family occupation background*		
Agriculture	296	67
Construction	63	14
Service industry	24	5
Business	15	3
Transport	16	5
Other	26	6

*Duration in current location (years)*		
< 5	365	83
> 5	75	17
Mean ± SD	3 ± 2	

*Monthly family income (USD)*		
< 100	361	82
100–200	68	15
> 200	11	3
Mean ± SD	50 ± 66	

**Table 2:** Knowledge about HIV/AIDS.

Questions	Correct responses *n* (%)	Wrong responses *n* (%)
Have you heard of HIV/AIDS?	226 (51)	214 (49)
Can a healthy-looking person be infected with HIV/AIDS?	230 (52)	210 (48)
Can HIV/AIDS be transmitted by mosquito bites?	109 (25)	331 (75)
Can HIV/AIDS be transmitted by sharing a meal with someone who is infected?	65 (15)	375 (85)
Can the risk of HIV transmission be reduced by having sex with only one faithful uninfected partner?	130 (30)	310 (70)
Can a person reduce the risk of getting HIV by using a condom every time they have sex?	121 (28)	319 (72)
Can a person get HIV by sharing sharp objects like razors/needles with someone who is infected?	354 (80)	86 (20)
Can a pregnant woman infected with HIV transmit the virus to her unborn child?	257 (58)	183 (42)
Can a woman with HIV transmit the virus to her newborn child through breastfeeding?	333 (76)	107 (24)
Is there a cure for HIV/AIDS?	110 (25)	330 (75)

**Table 3:** Differences in knowledge and attitude scores by gender and age groups.

Variable	*F*-statistic	*p* Value
Knowledge (gender)	0.222	0.638
Knowledge (age groups)	0.548	0.578
Attitude (gender)	0.164	0.685
Attitude (age groups)	2.067	0.128

**Table 4:** Attitudes toward people living with HIV/AIDS.

Questions	Yes *n* (%)	No *n* (%)
If a relative of yours became ill with HIV, would you be willing to care for him/her in your household?	150 (34)	290 (66)
If a student has HIV but is not sick, should he/she be allowed to continue attending school?	82 (19)	358 (81)
If a teacher has HIV but is not sick, should he/she be allowed to continue teaching in school?	90 (20)	350 (80)
Would you buy fresh vegetables from a shopkeeper or vendor if you knew that this person had HIV?	50 (11)	390 (89)
If a member of your family became infected with HIV, would you want it to remain a secret?	141 (32)	299 (68)

**Table 5:** Practices related to HIV/AIDS.

Questions	Yes *n* (%)	No *n* (%)
Have you ever been tested for HIV?	2 (0.45)	438 (100)
If yes, are you aware of your current HIV status?	2 (100)	0.0 (0.0)
Do you use injectable drugs?	24 (5)	416 (95)
If yes, do you share needles or syringes to inject drugs?	9 (39)	15 (61)
Have you ever had sexual intercourse?	92 (21)	348 (79)
If yes, did you use a condom during sexual intercourse?	2 (2)	90 (98)

## Data Availability

The data used in this study are available from the corresponding authors.

## References

[1] Fana T. (2021). Knowledge, Attitude and Practices Regarding HIV and AIDS Among High School Learners in South Africa. *The Open AIDS Journal*.

[2] Chiguzo A. N., Waweru R., Wangenya E. M. (2022). Assessment of Knowledge, Attitudes and Practices Regarding HIV/AIDS Among Road Construction Workers and Adjacent Communities in Kwale County. *International Research Journal of Multidisciplinary Scope*.

[3] (2024). Unaids. Fact Sheet 2024-Latest Global and Regional HIV Statistics on the Status of the AIDS Epidemic.

[4] (2024). HIV Statistics-Global and Regional Trends-UNICEF DATA. https://data.unicef.org/topic/hivaids/global-regional-trends/.

[5] (2025). Somalia-Somali Health and Demographic Survey 2020. NBS 2020. https://microdata.nbs.gov.so/index.php/catalog/50.

[6] (2024). LIVELIHOODS LOST Findings From Two Rounds of the Somalia Displacement Phone Survey (2022) 2. Join Data Center. https://www.jointdatacenter.org/wp-content/uploads/2024/11/Somalia-displacement-report_FINAL.pdf.

[7] Christane N. A., Roger Z. M., Masika J., Zhang Y., Liang Z. (2014). HIV/AIDS Prevalence, Knowledge, Attitudes and Related Behaviors Among Young People in Libreville, Gabon. *IOSR Journal of Humanities and Social Science*.

[8] Masoda M., Govender I. (2013). Knowledge and Attitudes About and Practices of Condom Use for Reducing HIV Infection Among Goma University Students in the Democratic Republic of Congo. *Southern African Journal of Epidemiology and Infection*.

[9] Melo J. S., Mittal M. L., Horyniak D., Strathdee S. A., Werb D. (2018). Injection Drug Use Trajectories Among Migrant Populations: A Narrative Review. *Substance Use & Misuse*.

[10] Dzah S. M., Tarkang E. E., Lutala P. M. (2019). Knowledge, Attitudes and Practices Regarding HIV/AIDS Among Senior High School Students in Sekondi-Takoradi Metropolis, Ghana. *African Journal of Primary Health Care & Family Medicine*.

[11] Durowade K. A., Babatunde O. A., Omokanye L. O. (2017). Early Sexual Debut: Prevalence and Risk Factors Among Secondary School Students in Ido-Ekiti, Ekiti State, South-West Nigeria. *African Health Sciences*.

[12] Leung Soo C., Pant Pai N., Bartlett S. J. (2023). Socioeconomic Factors Impact the Risk of HIV Acquisition in the Township Population of South Africa: A Bayesian Analysis. *PLOS Global Public Health*.

[13] Organization W. H. (2021). Violence Against Women Prevalence Estimates, 2018: Global, Regional and National Prevalence Estimates for Intimate Partner Violence Against Women and.

[14] Mabaso M., Makola L., Naidoo I., Mlangeni L. L., Jooste S., Simbayi L. (2019). HIV Prevalence in South Africa Through Gender and Racial Lenses: Results from the 2012 Population-based National Household Survey. *International Journal for Equity in Health*.

[15] Bile K., Warsame M., Ahmed A. D., Tlancet (2022). Fragile States Need Essential National Health Research: the Case of Somalia. *Lancet Global Health*.

[16] Stone J., Artenie A., Hickman M. (2022). The Contribution of Unstable Housing to HIV and Hepatitis C Virus Transmission Among People Who Inject Drugs Globally, Regionally, and at Country Level: A Modelling Study. *The Lancet Public Health*.

[17] Kovalenko G., Yakovleva A., Smyrnov P. (2023). Phylodynamics and Migration Data Help Describe HIV Transmission Dynamics in Internally Displaced People Who Inject Drugs in Ukraine. *PNAS Nexus*.

[18] Tanaka Y., Kunii O., Hatano T., Wakai S. (2008). Knowledge, Attitude, and Practice (KAP) of HIV Prevention and HIV Infection Risks Among Congolese Refugees in Tanzania. *Health & Place*.

[19] Vasylyeva T. I., Bojorquez I., Duc Pham M. (2022). Left Behind on the Path to 90-90-90: Understanding and Responding to HIV Among Displaced People. *Journal of the International AIDS Society*.

[20] Chynoweth S. K., Buscher D., Martin S., Zwi A. B. (2022). Characteristics and Impacts of Sexual Violence Against Men and Boys in Conflict and Displacement: a Multicountry Exploratory Study. *Journal of Interpersonal Violence*.

[21] Chen M. I., von Roenne A., Souare Y. (2008). Reproductive Health for Refugees by Refugees in Guinea II: Sexually Transmitted Infections. *Conflict and Health*.

[22] Herek G. M., Capitanio J. P., Widaman K. F. (2002). HIV-Related Stigma and Knowledge in the United States: Prevalence and Trends, 1991-1999. *American Journal of Public Health*.

[23] (2023). Banadir IDP Site Verification-November 2023.

[24] Imene A. (2023). Impact of Performance Evaluation System on Employee Performance in Nigeria Local Government Administration: A Study of Ukwuani Local Government Administration of Delta State Nigeria. *Journal of Social Sciences and Management Studies*.

[25] Nubed C. K., Akoachere J.-F. T. K. (2016). Knowledge, Attitudes and Practices Regarding HIV/AIDS Among Senior Secondary School Students in Fako Division, South West Region, Cameroon. *BMC Public Health*.

[26] Bamise O. F., Bamise C. T., Adedigba M. A. (2014). Knowledge About HIV/AIDS Among Nigerian Adolescents in the 21st Century: A Cross-Sectional Study. *Radiance Research Academy*.

[27] Sohn A., Park S. (2012). HIV/AIDS Knowledge, Stigmatizing Attitudes, and Related Behaviors and Factors that Affect Stigmatizing Attitudes Against HIV/AIDS Among Korean Adolescents. *Osong Public Health and Research Perspectives*.

[28] Adeboye A., Yongsong Q., Akinwumi O., James N. (2016). Knowledge, Attitude and Practices of HIV/AIDS Among High School Students in Eastern Cape, South Africa. *Journal of Human Ecology*.

[29] Directorate of National Statistics (2020). The Somali Health and Demographic Survey. *Federal Government of Somalia*.

[30] Mohamud A. K., Ahmed O. A., Mohamud A. A., Dirie N. I. (2023). Prevalence of and Factors Associated With Depression Among Adult Patients Living With HIV/AIDs Undergoing ART Unit in Banadir Hospital, Mogadishu Somalia. *BMC Psychiatry*.

